# Induction of caspase-dependent extrinsic apoptosis by apigenin through inhibition of signal transducer and activator of transcription 3 (STAT3) signalling in HER2-overexpressing BT-474 breast cancer cells

**DOI:** 10.1042/BSR20150165

**Published:** 2015-12-22

**Authors:** Hye-Sook Seo, Jae Kyung Jo, Jin Mo Ku, Han-Seok Choi, Youn Kyung Choi, Jong-Kyu Woo, Hyo in Kim, Soo-yeon Kang, Kang min Lee, Koong Won Nam, Namkyu Park, Bo-Hyoung Jang, Yong Cheol Shin, Seong-Gyu Ko

**Affiliations:** * Laboratory of Clinical Biology and Pharmacogenomics and Center for Clinical Research and Genomics, College of Korean Medicine, Kyung Hee University, 26 Kyungheedae-ro, Dongdaemun-gu, Seoul 130-701, Republic of Korea; † Next-generation Pharmaceutical Research Center, Korea Institute of Toxicology, 141 Gajeong-ro, Yuseong-gu, Daejeon 305-343, Republic of Korea; ‡ College of Pharmacy, Gachon University of Medicine and Science, 7-45 Songdo-dong, Yeonsu-gu, Incheon 406-840, Republic of Korea

**Keywords:** apigenin, apoptosis, breast cancer, HER2, p53, STAT3

## Abstract

Phytoestrogen intake is known to be beneficial to decrease breast cancer incidence and progression. But its molecular mechanisms of action are still unknown. The present study aimed to examine the effect of apigenin on proliferation and apoptosis in HER2-expressing breast cancer cells. In our experiments, apigenin inhibited the proliferation of BT-474 cells in a dose- and time-dependent manner. Apigenin also inhibited clonogenic survival (anchorage-dependent and -independent) of BT-474 cells in a dose-dependent manner. These growth inhibitions were accompanied with an increase in sub-G_0_/G_1_ apoptotic populations. Apigenin-induced extrinsic a caspase-dependent apoptosis up-regulating the levels of cleaved caspase-8 and cleaved caspase-3, and inducing the cleavage of poly (ADP-ribose) polymerase (PARP). Whereas, apigenin did not induce apoptosis via intrinsic mitochondrial apoptosis pathway since this compound did not decrease mitochondrial membrane potential without affecting the levels of B-cell lymphoma 2 (Bcl-2) and Bcl-2-associated X protein (BAX). Apigenin reduced the expression of phospho-JAK1, phospho-JAK2 and phospho-STAT3 and decreased signal transducer and activator of transcription 3 (STAT3) dependent luciferase reporter gene activity in BT-474 cells. Apigenin inhibited CoCl_2_-induced VEGF secretion and decreased the nuclear translocation of STAT3. Our study indicates that apigenin induces apoptosis through inhibition of STAT3 signalling and could serve as a useful compound to prevent or treat HER2-overexpressing breast cancer.

## Abstract (precis)

Apigenin induced caspase-dependent extrinsic apoptosis through inhibition of STAT3 signaling in HER2 overexpressing BT-474 breast cancer cells.

## INTRODUCTION

Phytoestrogens are a large group of plant-derived compounds and contain a phenolic ring which allows them to bind to the oestrogen receptor (ER), showing weak oestrogenic activity [1]. There are five major classes of phytoestrogens: flavones, isoflavones, lignans, coumestans and stilbenes. Apigenin (4',5,7,-trihydroxyflavone) is one of the flavones found in various foods consumed by humans. Apigenin is found in many fruits and vegetables such as celery, parsley, Chinese cabbage, bell peppers, cherries, apples and grapes. It is also found in wine and tea, including chamomile. Like most flavones, apigenin has anti-inflammatory [2], antioxidant [3], anti-telomerase [4] and antidepressant [5] properties. The most important research has been for its potential to fight cancer. Epidemiologic studies indicate that a diet rich in apigenin is associated with a decreased risk of certain cancers, particularly cancers of the breast, digestive tract, skin, prostate, lung, ovary and certain haematological malignancies [6–11]. In *in vivo* models, apigenin suppressed prostate tumorigenesis in transgenic adenocarcinoma of the mouse prostate (TRAMP) mice through the PI3K/Akt/FoxO-signalling pathway [12]. Administration of apigenin resulted in attenuation of tumour growth in U937 xenografts accompanied by inactivation of Akt and activation of JNK [13]. Apigenin significantly inhibited tumour growth in nude mice suppressing HIF-1α and VEGF expression [14]. In *in vitro* models, apigenin-induced growth inhibition and apoptosis in a variety of cancer cell lines including breast [15], lung [16], colon [17,18], prostate [19], leukaemia [20] and pancreatic [21] cells. These studies suggest that apigenin could be developed as a chemopreventive and/or chemotherapeutic agent for cancer.

Apoptosis is a form of cell death in which a programmed sequence of events leads to the elimination of cells without releasing harmful substances into the surrounding area [2]. Apoptosis is considered a vital component of various processes including normal cell turnover, proper development and functioning of the immune system, hormone-dependent atrophy, embryonic development and chemical-induced cell death [22]. Inappropriate apoptosis can play a role in many diseases including neurodegenerative diseases, ischemic damage, autoimmune disorders and many types of cancer [22]. Two core pathways exist to induce apoptosis, the extrinsic–death receptor pathway and intrinsic–mitochondrial pathway [23]. The extrinsic pathway is related to the activation of the death receptors, such as Fas and tumour necrosis factor receptors (TNFR). Death domains (DD) of Fas are oligomerized and recruit Fas-associated death domain (FADD) and procaspase-8 to form death-inducing signalling complex (DISC). Procaspase-8 is cleaved and activated and released from the DISC into the cytoplasm where it activates caspase-3 to induce apoptosis [24,25]. The intrinsic pathway is related to changes in mitochondrial membrane potential (ΔΨm) and mitochondrial permeability transition, resulting in mitochondrial release of apoptogenic factors such as cytochrome *c* and apoptosis-inducing factor (AIF) into the cytoplasm [26]. Cytochrome *c* binds to APAF1 and recruits procaspase-9 to form an apoptosome; caspase-9 activates effector caspases such as caspase-3 to induce apoptosis [27]. Caspase-3 from both extrinsic and intrinsic pathways is responsible for the cleavage of poly (ADP-ribose) polymerase (PARP) during cell death [28].

Breast cancers with human epidermal growth factor receptor (HER2) gene amplification or HER2 protein overexpression are called HER2-positive [29]. Approximately 20% of breast cancer cases are HER2-positive [29]. HER2-positive breast cancers tend to be more aggressive than other types of breast cancer [30]. They are also less responsive to hormone treatment [31]. However, treatments that specifically target HER2 exist: trastuzumab (herceptin) and lapatinib (tykerb). Trastuzumab binds to domain IV of the extracellular segment of the HER2 and induces cell growth arrest during the G1 phase of the cell cycle resulting in reduced proliferation [32,33]. Trastuzumab induces some of its effect by down-regulation of HER2/neu leading to disruption of receptor dimerization and signalling through the downstream PI3K cascade [34]. Lapatinib inhibits the tyrosine kinase activity associated with HER2 [35]. Lapatinib decreases tumour-causing breast cancer stem cells [36]. Lapatinib inhibits receptor signal processes by binding to the ATP-binding pocket of the HER2 protein kinase domain, preventing self-phosphorylation and subsequent activation of the signal mechanism [37]. However, many women do not respond to these drugs or develop resistance [38]. This has resulted in significant efforts to find other compounds which could effectively treat HER2-overexpressing breast cancer.

In the present study, we investigated whether apigenin displays growth-suppressive activity on HER2-overexpressing breast cancer cells. For this purpose, we tested the effects of apigenin on proliferation and apoptosis of BT-474 cells; we performed proliferation assay, MTT assay and FACS analysis to evaluate the cytotoxicity of apigenin in breast cancer cells. We also investigated the mechanism by which apigenin regulates the growth of BT-474 cells analysing the cell cycle and measuring the levels of apoptotic molecules and intracellular signalling molecules. We also verified whether apigenin inhibits signal transducer and activator of transcription 3 (STAT3) signalling pathway, leading to growth suppression of HER2-expressing breast cancer cells. Since we report here that apigenin may suppress HER2-positive breast cancer, the present study advances human health.

## MATERIALS AND METHODS

### Compounds

Apigenin (4',5,7-trihydroxyflavone), carbonyl cyanide 4-(trifluoromethoxy) phenylhydrazone (FCCP) and HIF-1α inhibitor (EF-24) were purchased from Sigma Chemical Co. These compounds were dissolved in dimethyl sulfoxide (DMSO) and the final concentration of DMSO in the controls and each sample did not exceed 0.1%. We found that 0.1% DMSO did not affect the cell growth rate compared with 0% DMSO (no treatment) in breast cancer cells (data not shown). JC-1 was obtained from Molecular Probes (Invitrogen). The caspase-8 inhibitor Z-IETD-*fmk* and the caspase-9 inhibitor Z-LEHD*-fmk* were obtained from R&D Systems, Inc. The STAT3 inhibitor S3I-201 was obtained from Calbiochem. The JAK inhibitor I was purchased from Santa Cruz Biotechnology, Inc. An EZ-western chemiluminescent detection kit was purchased from Daeillab Service Co.

### Cell cultures

BT-474 human breast cancer cells (A.T.C.C.) were cultured in RPMI 1640 medium containing 50 U/ml penicillin, 50 mg/ml streptomycin and 10% foetal bovine serum (FBS; Welgene) at 37°C in an atmosphere of 5% CO_2_.

### Antibodies

Primary antibodies directed against FAS, cleaved caspase-8, caspase-3, cleaved caspase-3 and PARP were purchased from Cell Signaling Technology, Inc. Primary antibodies directed against B-cell lymphoma 2 (Bcl-2), Bcl-2-associated X protein (BAX), p53, phospho-p53 (Ser^15^) and VEGF were obtained from Santa Cruz Biotechnology, Inc. Primary antibodies directed against STAT3, phospho-STAT3 (Tyr^705^) phospho-JAK1 (Tyr^1022^/Tyr^1023^) and phospho-JAK2 (Tyr^1007^/Tyr^1008^) were obtained from Upstate-Millipore. Primary antibody against HIF-1α was purchased from BD Biosciences. The anti-tubulin antibody was from Sigma Chemical Co. Horseradish peroxidase (HRP)-conjugated secondary antibodies (mouse and rabbit) were purchased from Calbiochem and anti-goat secondary antibody was from Jackson ImmunoResearch.

### Cell proliferation assay

Cells were seeded in 12-well culture plates at a density of 5×10^4^ cells/well. After the cells were exposed to different concentrations of apigenin (0–60 μM) and incubated for 3 days, they were harvested by trypsinization, resuspended in 1–2 ml of medium, and counted using a haemocytometer.

### MTT assay

Cells were seeded in 96-multiwell culture plates at a density of 3×10^3^ cells/well and incubated for 24 h at 37°C. Then, they were treated with different concentrations of apigenin (0–60 μM) for 24 h, 48 h or 72 h. After incubation, MTT reagents (0.5 mg/ml) were added to the each well and the plates were incubated in the dark at 37°C for 2 h. At the end of the incubation, the medium was removed, the resulting formazan was dissolved in DMSO and the optical density was measured at 570 nm using an ELISA plate reader.

### Clonogenic survival assays (anchorage-dependent and anchorage-independent)

For anchorage-dependent colony formation assay, cells were seeded into six-well culture plates at a density of 5×10^2^ cells/well. After overnight incubation, they were treated with different concentrations of apigenin (0–60 μM) or vehicle and maintained for 10 days at 37°C. Cells were fed every 3 days by removing old medium and adding fresh medium containing apigenin. Finally, plates were stained with haematoxylin and the colony number was counted. For anchorage-independent colony formation assay, soft agar was used. 1×10^3^ cells were suspended in 1 ml of 0.6% soft agar that was layered on top of 1 ml of 1% solidified agar in each well of 12-well plates. The plates were then incubated for 15–21 days in complete RPMI medium containing apigenin (0–60 μM). The medium was changed every 3 days during this period. At the end of experiment, tumour cell colonies measuring at least 30 μm were counted under using a dissection microscope.

### Cell-cycle analyses by flow cytometry

Cells were harvested with 0.25% trypsin and washed once with phosphate buffered saline (PBS). After centrifugation (1500 ***g***), the cells were fixed in cold 95% ethanol with 0.5% Tween-20, and stored at -20°C for at least 30 min. The cells were incubated in 50 μg/ml of propidium iodide (PI) (including 1% of sodium citrate and 50 μg/ml of RNase A) at room temperature in the dark for 30 min. The analysis of apoptotic cells was performed with a FACScan flow cytometer (Becton Dickinson) and the data were analysed using CellQuest software.

### Analysis of mitochondrial transmembrane potential (ΔΨm)

Cells were seeded at a density of 1×10^6^ cells/dish in 100 mm dishes and incubated for 24 h at 37°C. After stabilization, the cells were treated with apigenin (0–60 μM) and vehicle for 72 h. After harvest by treatment with trypsin-EDTA, the cells were washed with cold PBS, centrifuged at 1500 ***g*** for 5 min and stained with 4 μg/ml JC-1 for 15 min at 37°C in the dark. The data were analysed by FACSCalibur flow cytometry (BD Biosciences) measuring the green fluorescence and red fluorescence at 514/529 nm (FL-1) and 585/590 nm (FL-2), respectively.

### Western blot analysis

Cells were lysed in modified RIPA buffer (150 mM NaCl, 1% NP-40, 0.5% deoxycholate, 0.1% SDS, 50 mM Tris (pH 8.0), 1 mM EDTA, 1 mM phenylmethylsulfonyl fluoride (PMSF), 1 mM NaF, 1 mM Na_3_VO_4_ and protease inhibitor mixture). The lysates were cleared by centrifugation at 13000 ***g*** for 15 min and the supernatants were collected. The protein concentration was quantified using a Bio-Rad Bradford protein assay (Bio-Rad Laboratories). Equal amounts of protein lysates were used for western blot analysis with the indicated antibodies. Immunoreactive protein bands were detected with an EZ-Western Detection kit (Daeillab Service Co.).

### Immunocytochemistry

Cells (2×10^4^ cells/well) were seeded in eight-well chamber slides, incubated for 24 h at 37°C and treated with apigenin (60 μM) in the presence or absence of CoCl_2_ for another 24 h. The cells were fixed with 4% paraformaldehyde for 30 min and treated with 3% hydrogen peroxide (H_2_O_2_) in methanol for 20 min to quench endogenous peroxidase activity. The cells were washed with PBS, blocked with 5% BSA in PBS for 1 h and incubated with the anti-STAT3 primary antibody (1:100 dilution) overnight at 4°C. After washing with PBS, the cells were incubated with anti-rabbit biotin-conjugated secondary antibody for 1 h at room temperature. Then, the cells were treated with Vectastain ABC reagent (Vector Laboratories) for 30 min at 4°C and stained with diaminobenzidine tetra chloride (DAB) and haematoxylin. The cells were mounted with mounting medium and subsequently analysed by microscopy.

### RNA extraction and reverse transcription-polymerase chain reaction (RT-PCR)

Whole-cell lysates under diverse conditions were prepared by washing with ice-cold PBS. Total RNA was isolated using Trizol reagent (iNtRON Biotechnology). Total RNA was treated with 2 units of RNase-free DNase at 37°C for 30 min, extracted with phenol/chloroform/isoprophanol, and precipitated with ethanol. The RNA concentration was determined by measuring the absorbance at 260 nm using a nanodrop, and the ratio of absorbance at 260 and 280 nm was 1.8 or higher. cDNA was synthesized from 2 μg of total RNA as a template in 20 μl reaction mixture containing 5X first strand buffer, 0.1 M DTT, 10 mM dNTP and 200 unit M-MLV reverse transcriptase (iNtRON biotechnology). cDNA was incubated at 42°C for 1 h and inactivated at 95°C for 5 min. After inactivation, the cDNA was stored at -20°C until use. RT-PCR was performed by co-amplification of the genes with a reference gene by use of the cDNA template and corresponding gene-specific primer sets. The primer sequences are shown in Table 1. PCR was conducted out in a total volume of 25 μl containing 5 μl of cDNA solution, 25 μM of sense primers, and 25 μM of antisense primers, 1X PCR buffer, 2.5 mM MgCl_2_ and 2.5 unit Taq DNA polymerase (Takara Korea, Seoul, Korea). The sequencing involved 30 cycles at 94°C, 45 s for denaturation, 58°C, 45 s for annealing, and 72°C, 45 s for extension. The resulting PCR products were resolved on 1% agarose gels containing ethidium bromide.

**Table 1:** Primer sequences RT-PCR was performed by co-amplification of the genes with a reference gene by use of the cDNA template and corresponding gene-specific primer sets.

Primers	Sequence
HIF-1α	Forward 5' TCA CCA CAG GAC AGT ACA GGA TGC 3'
	Reverse 5' CCA GCA AAG TTA AAG CAT CAG GTT CC 3'
VEGF	Forward 5' AAG GCC CAC AGG GAT TTT CT 3'
	Reverse 5' AGG AGG GCA GAA TCA TCA CG 3'
GAPDH	Forward 5' CGG CCA TCA CGC CAC AGT TT 3'
	Reverse 5' CGT CTT CAC CAC CAT GGA GA 3'

### Measurement of VEGF and MMP-9 secreted from BT-474 cells by ELISA

To assess the level of VEGF and MMP-9 in the BT-474 cell supernatants, the cells were treated with apigenin (0–60 μM) in the presence or absence of CoCl_2_ (100 μM) to mimic hypoxia. After 24 h, the media were collected, centrifuged (1500 ***g***) to remove the cellular debris, and stored at -70°C until assayed for VEGF and MMP-9. The amount of VEGF and MMP-9 secreted into the culture medium was measured by ELISA according to the manufacturer's instructions (R&D Systems). Briefly, 96-well plates were coated with capture antibody in ELISA coating buffer and incubated overnight at 4°C. The plates were then washed with PBS with 0.05% Tween 20 (PBS-T) and subsequently blocked with 10% FBS in PBS for 1 h at 20°C. Serial dilutions of standard antigen or sample in dilution buffer (10% FBS in PBS) were added to the plates, and the plates were incubated for 2 h at 20°C. After washing, biotin-conjugated anti-mouse IgE and streptavidin-conjugated horseradish peroxidase (SAv-HRP) were added to the plates, and the plates were incubated for 1 h at 20°C. Finally, the tetramethylbenzidine (TMB) substrate was added to the plates, and after 15 min of incubation in the dark, 2N H_2_SO_4_ was added to stop the reaction. The optical density was measured at 450 nm on an automated ELISA reader.

### STAT3 luciferase reporter assay

BT-474 cells were plated and allowed to attach by overnight incubation at 37°C. Cells were transiently transfected with p4xM67-TK-luc plasmid (Addgene plasmid 8688, Addgene) containing four copies of the STAT-binding site (TTCCCGTAA). The next day, cells were treated with different concentrations of apigenin (0–60 μM) for 24 h and then submitted to the luciferase assays. Luciferase assays were performed using a dual-luciferase assay kit according to the manufacturer's instructions (Promega). Finally, luciferase activities were determined using a luminometer (BMG Labtech).

### Statistical analysis

All experiments were performed in triplicate. The data for the cell proliferation assay, MTT assay, ELISA assay and STAT3 luciferase reporter assay are expressed as the mean ± standard deviation (S.D.). The standard deviations for all of the measured biological parameters are displayed in the appropriate figures. A Student's *t*-test was used for single variable comparisons, and a *P*-value of <0.05 was considered statistically significant.

## RESULTS

### Apigenin suppresses the growth of BT-474 cells

**Figure 1.**
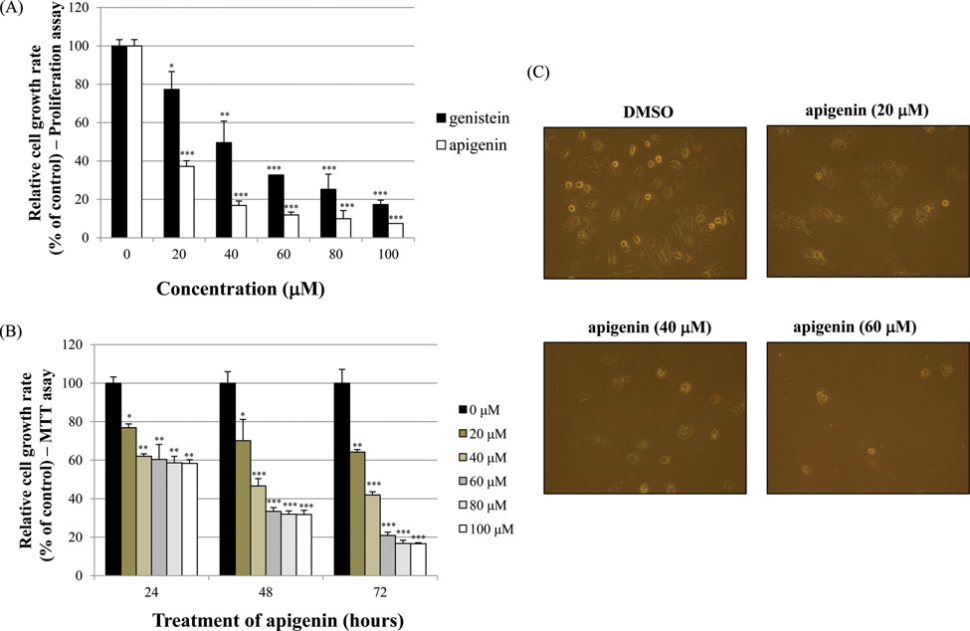
Effect of apigenin on BT-474 cell growth (**A**) BT-474 cells were treated with different doses of apigenin and genistein (0–100 μM). After 72 h, cell viability was assessed using a cell proliferation assay. (**B**) BT-474 cells were treated with different doses of apigenin (0–100 μM). The relative cell growth rate was measured by MTT assay after 24 h, 48 h and 72 h. The growth rate of the vehicle-treated cells was set to 100%, and the relative decrease in cell viability resulting from the phytoestrogen treatment was expressed as a percentage of the control. (**C**) BT-474 cells were treated with different doses of apigenin (0–60 μM) for 72 h and photographed by phase-contrast microscopy (original magnification, ×40). Control cells were treated with DMSO alone. Data are shown as the means of three independent experiments (error bars denote S.D.). *P<0.05, **P<0.01, ***P<0.001.

The effects of apigenin on cell growth were measured by cell proliferation assay and MTT assay in BT-474 cells. As shown in Figure 1(A), apigenin and genistein significantly inhibited BT-474 cell proliferation in a dose-dependent manner (0–100 μM) after 72 h of treatment (proliferation assay). Between two phytoestrogens, apigenin had the stronger growth-suppressive activity compared with genistein in BT-474 cells. Therefore, we chose apigenin for our experimental study. In addition, the time-dependent growth-suppressive activity of apigenin was measured by the MTT assay, as shown in Figure 1(B). It seems that the proliferation assay was more sensitive than the MTT assay with respect to measuring the intensity of the cell growth inhibition, as shown in Figures 1(A) and 1(B). Moreover, the growth inhibition induced by apigenin was verified by microscopic observation. The results in Figure 1(C) show that apigenin effectively inhibited the growth rate of BT-474 monolayer cells after 72 h of treatment. Of note, apigenin also induced morphological changes in these cells (Figure 1C).

### Apigenin inhibits clonogenic survival of BT-474 cells

**Figure 2:**
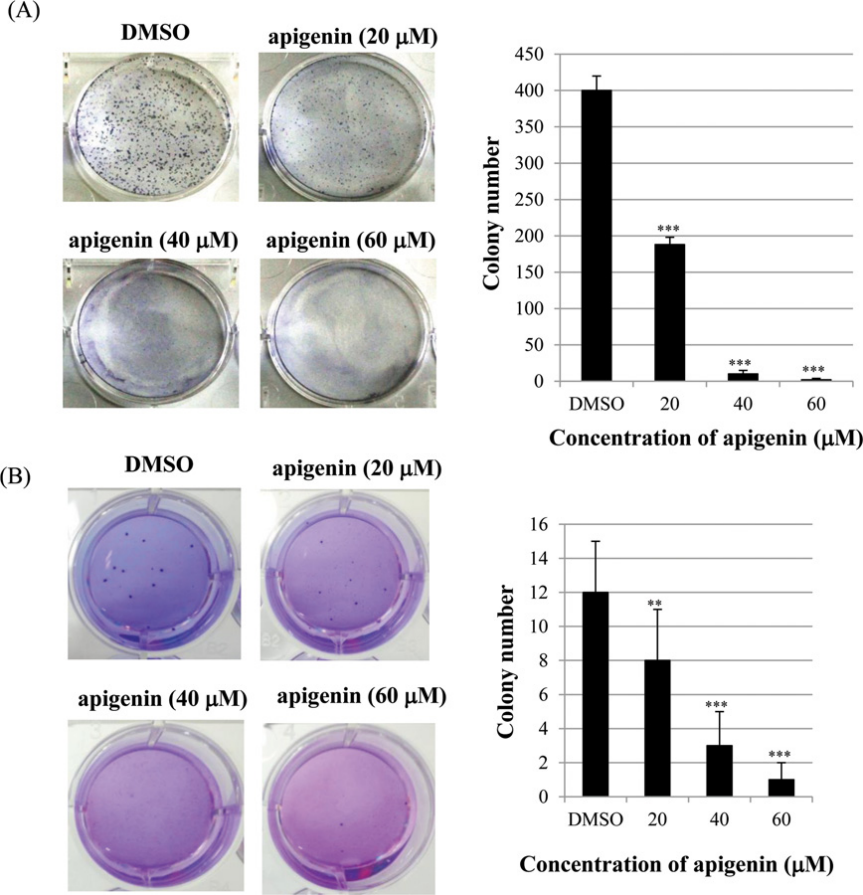
Apigenin inhibits anchorage-dependent and anchorage-independent clonogenic survival of BT-474 cells (**A**) BT-474 cells were seeded into six-well culture plates at a density of 5×10^2^ cells/well. After overnight incubation, cells were treated with different concentrations of apigenin (0–60 μM) and maintained for 10 days at 37°C. Finally, plates were stained with haematoxylin and the colony number was counted. (**B**) 1×10^3^ cells were suspended in 1 ml of 0.6% soft agar that was layered on top of 1 ml of 1% solidified agar in each well of 12-well plates. The plates were then incubated for 15–21 days in complete RPMI medium containing apigenin. Colonies were stained with crystal violet.

Next, we investigated the effect of apigenin on clonogenic survival of BT-474 cells using clonogenic survival assays (anchorage-dependent and anchorage-independent). As shown in Figure 2(A), apigenin significantly inhibited anchorage-dependent colony formation dose-dependently in BT-474 cells. Consistently with this result, apigenin strongly decreased anchorage-independent colony formation in BT-474 cells (Figure 2B). These results suggest that apigenin inhibits clonogenic survival of BT-474 cells.

### The growth-suppressive activity of apigenin is accompanied by an increase in the sub-G_0_/G_1_ apoptotic population in BT-474 cells

**Figure 3:**
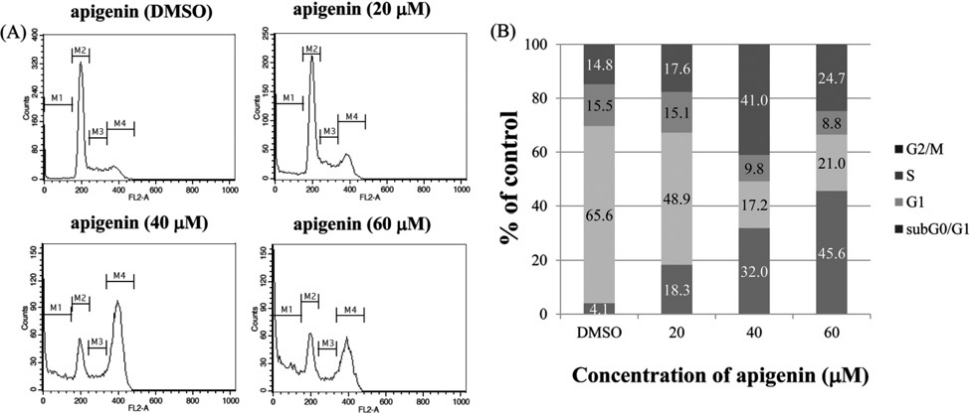
Effect of apigenin on the cell cycle and sub-G_0_/G_1_ apoptotic population of BT-474 cells (**A**) BT-474 cells were treated with apigenin (0–60 μM) and fixed 72 h later for flow cytometry. Propidium iodide-labelled nuclei were analysed for DNA content. (**B**) The sub-G_0_/G_1_ apoptotic population and the G_1_, S and G_2_/M phase populations were quantified using DNA histograms. The data shown are representative of three independent experiments that gave similar results.

To investigate whether apigenin inhibits cell proliferation by promoting changes in cell-cycle progression, the effect of apigenin on the cell-cycle profile was assessed in BT-474 cells. For this purpose, cells were treated with apigenin (0–60 μM) for 72 h and then analysed for cell-cycle stage by flow cytometry. The results demonstrated that apigenin induced an increase in the sub G_0_/G_1_ apoptotic population in BT-474 cells (Figure 3).

### Apigenin induces apoptosis via a caspase-dependent pathway in BT-474 cells

**Figure 4:**
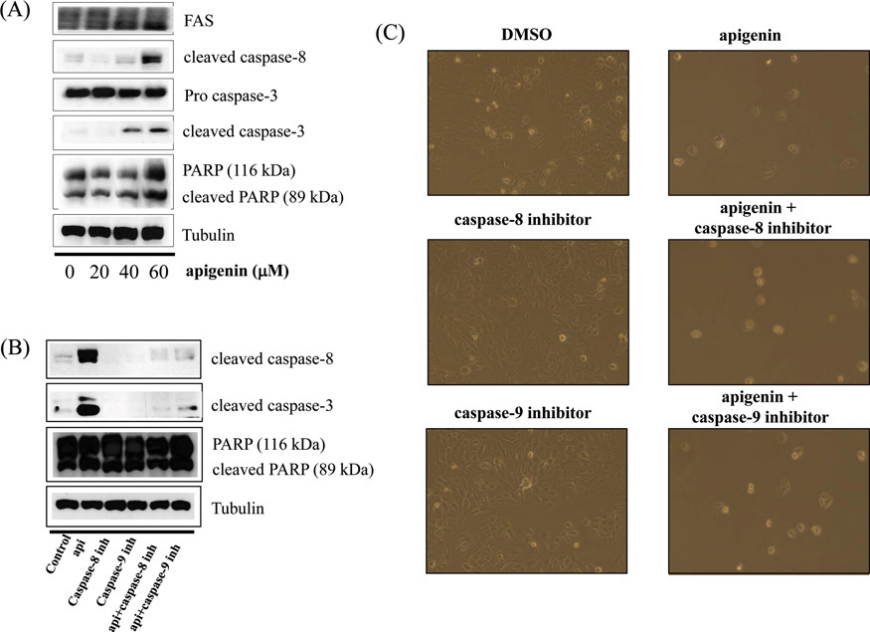
Apigenin induces a caspase-dependent apoptosis in BT-474 cells (**A**) Apigenin induces apoptosis via a caspase-dependent apoptosis pathway in BT-474 cells. BT-474 cells were treated with apigenin (0–60 μM) for 24 h. Whole-cell lysates were analysed by western blotting with anti-FAS, anti-cleaved caspase-8, anti-caspase-3, anti-cleaved caspase-3, anti-PARP and anti-tubulin antibodies. The data shown are representative of three independent experiments that gave similar results. (**B**) Effect of caspase-8 and caspase-9 inhibitors on apigenin-induced apoptosis in BT-474 cells. BT-474 cells were exposed to 60 μM apigenin with or without the caspase-8 inhibitor (40 μM) or the caspase-9 inhibitor (40 μM) for 24 h, the cell lysates were separated by SDS/PAGE, and western blotting with specific antibodies was performed (anti-cleaved caspase-8, anti-cleaved caspase-3, anti-PARP and anti-tubulin). The data shown are representative of three independent experiments that gave similar results. (**C**) Effect of caspase-8 and caspase-9 inhibitors on BT-474 cell proliferation. BT-474 cells were exposed to 60 μM apigenin with or without the caspase-8 inhibitor (40 μM) or the caspase-9 inhibitor (40 μM) for 72 h and photographed by phase-contrast microscopy (original magnification, ×40).

In this step, we investigated whether apigenin activates the caspase-dependent apoptosis pathway by measuring the expression of caspase-8, caspase-3 and PARP. We observed that apigenin up-regulated the levels of cleaved caspase-8 and caspase-3, and induced the cleavage of PARP in BT-474 cells (Figure 4A). This indicates that apigenin strongly promotes apoptosis via a caspase-dependent mechanism in BT-474 cells. We also found that the cleavage of caspase-8 and caspase-3 was inhibited by the caspase-8 inhibitor Z-IETD-*fmk* and the caspase-9 inhibitor Z-LEHD*-fmk* (Figure 4B), but apigenin prevented this inhibition and slightly induced the cleavage of caspase-8 and caspase-3 in the presence of Z-IETD-*fmk* and Z-LEHD*-fmk* (Figure 4B). Moreover, the caspase-8 and caspase-9 inhibitors did not suppress cell growth, but apigenin was able to induce apoptosis even in their presence (Figure 4C). These results confirm that apigenin strongly promotes apoptosis in BT-474 cells.

### Apigenin induces extrinsic apoptosis in BT-474 cells

**Figure 5:**
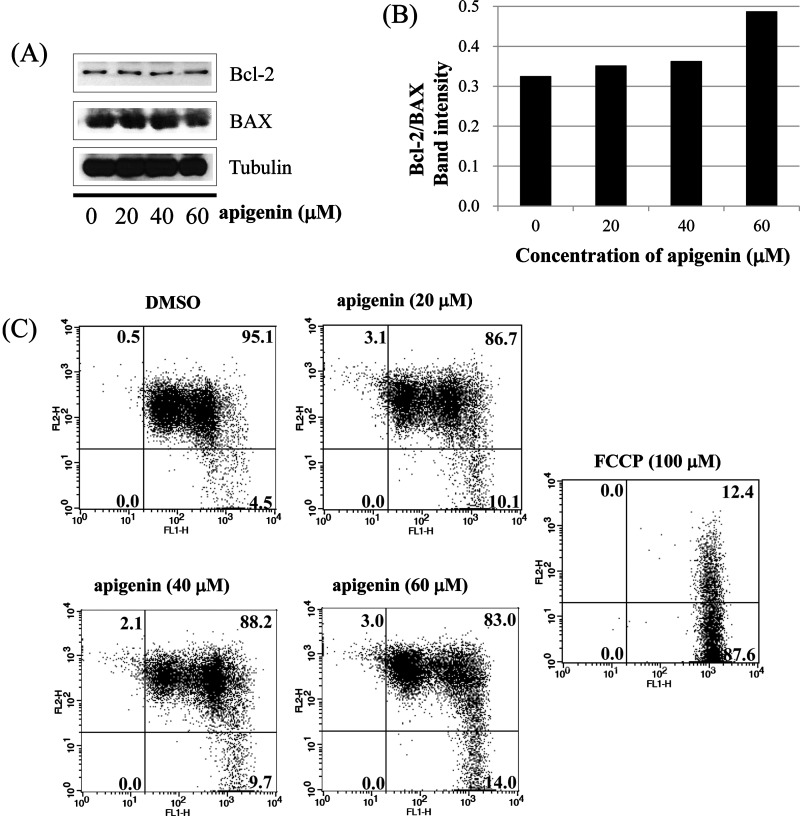
Apigenin induces apoptosis via extrinsic pathway in BT-474 cells (**A**) and (**B**) analysis of intrinsic apoptosis-related molecules. BT-474 cells were treated with apigenin (0–60 μM) for 24 h. Total proteins were analysed by western blotting with anti-Bcl-2, anti-BAX and anti-tubulin antibodies. (**C**) BT-474 cells were incubated with apigenin (0–60 μM) for 72 h and were dyed with JC-1 (4 μg/ml). The data were analysed by FACSCalibur flow cytometry measuring the green fluorescence and red fluorescence at 514/529 nm (FL-1) and 585/590 nm (FL-2), respectively. The data shown are representative of three independent experiments that gave similar results.

Next, we investigated whether apoptosis induced by apigenin occurs via extrinsic apoptosis pathway in BT-474 cells. For this purpose, we measured the levels of BCL2 family members (BAX and Bcl-2) which are important in non-extrinsic (intrinsic mitochondrial) apoptosis pathway. We found that apigenin failed to regulate the levels of BAX and Bcl-2 in BT-474 cells as shown in Figures 5(A) and 5(B). We also measured the loss of mitochondrial transmembrane potential (ΔΨm) within the cells using JC-1. JC-1 is able to selectively enter mitochondria and reversibly transforms colour from red to green when the membrane potential decreases. In non-apoptotic cells with high mitochondrial ΔΨm, JC-1 spontaneously forms complexes known as J-aggregates with intense red fluorescence. On the other hand, in apoptotic cells (especially mitochondria-mediated apoptotic cells) with low ΔΨm, JC-1 remains in the monomeric form, which shows only green fluorescence. In our study, apigenin did not induce a low mitochondrial transmembrane potential (ΔΨm), showing relatively weak green fluorescence (DMSO; 4.5%, Api 20 μM; 10.1%, Api 40 μM; 9.7%, Api 60 μM; 14.0%) compared with FCCP (positive control: 87.6%) (Figure 5C). These results demonstrate that apigenin does not induce apoptosis via the intrinsic mitochondrial pathway but induces apoptosis via the extrinsic pathway in BT-474 cells.

### Effect of apigenin on STAT3 activation in BT-474 cells

**Figure 6:**
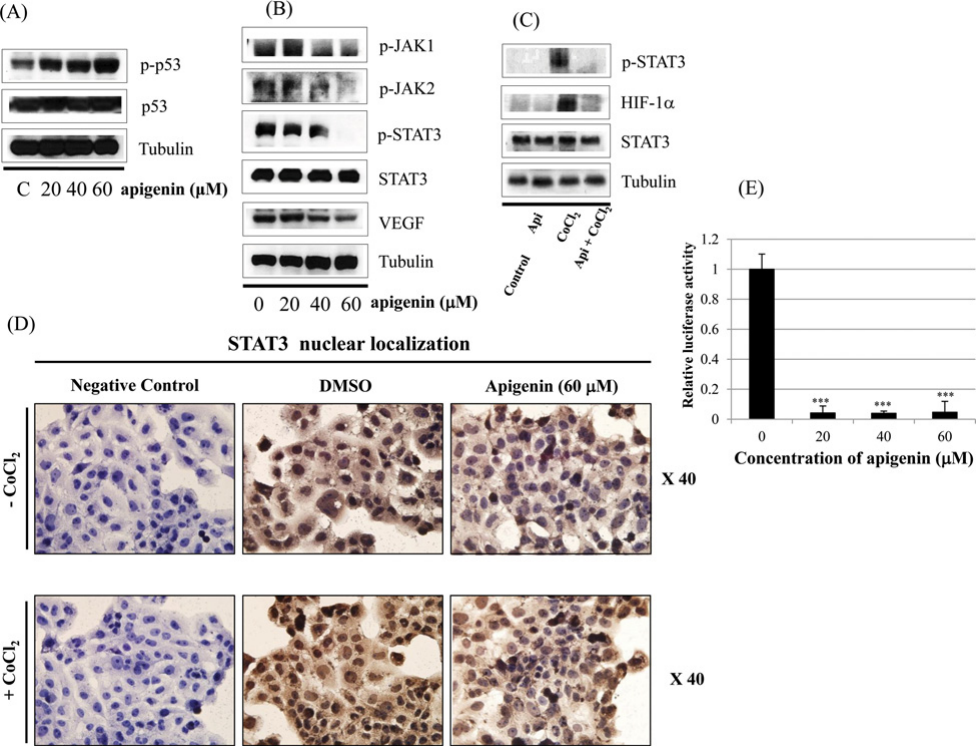
Effect of apigenin on STAT3 activation in BT-474 cells (**A**) BT-474 cells were treated with apigenin (0–60 μM) for 24 h. Whole-cell lysates were analysed by western blotting with anti-p-p53, anti-p53 and anti-tubulin antibodies. (**B**) BT-474 cells were treated with apigenin (0–60 μM) for 24 h. Whole-cell lysates were analysed by western blotting with anti-p-JAK1, anti-p-JAK2, anti-p-STAT3, anti-STAT3, anti-VEGF and anti-tubulin antibodies. (**C**) BT-474 cells were treated with apigenin (60 μM) for 24 h in the presence or absence of CoCl_2_ (4 h). Whole-cell lysates were analysed by western blotting with anti-phospho-STAT3, anti-HIF-1α, anti-STAT3 and anti-tubulin antibodies. (**D**) BT-474 cells were treated with apigenin (60 μM) for 24 h in the presence or absence of CoCl_2_ and then submitted to immunocytochemistry for detection of nuclear STAT3. The data shown are representative of three independent experiments that gave similar results. (**E**) BT-474 cells were transiently transfected with p4xM67-TK-luc plasmid containing four copies of the STAT-binding site, treated with apigenin (0–60 μM) and submitted to dual luciferase assay. Data are shown as the means of three independent experiments (error bars denote S.D.). *P<0.05, **P<0.01, ***P<0.001.

Figure 6(A) shows that apigenin up-regulates phospho-p53 (p-p53). In Figure 6(B), we investigated whether apigenin affects STAT3 signalling measuring levels of p-STAT3 and VEGF (STAT3 target gene). We found that apigenin reduced the expression of p-STAT3 as well as p-JAK1 and p-JAK2 (upstream kinases of STAT3) (Figure 6B). Apigenin also reduced the level of VEGF (Figure 6B). Since STAT3 is a potential modulator of HIF-1α, we observed the relationship between STAT3 and HIF-1α. We found that apigenin suppressed the expression of p-STAT3 and HIF-1α that was up-regulated by CoCl_2_ (hypoxia mimic) (Figure 6C). Immunocytochemical staining indicated that apigenin decreased the nuclear localization of STAT3 in the presence and absence of CoCl_2_ (Figure 6D). Figure 6(E) demonstrated that apigenin strongly decreased STAT3 transcriptional activity as revealed by transient transfection and luciferase assay.

### Effect of apigenin on STAT3 target genes in BT-474 cells

**Figure 7:**
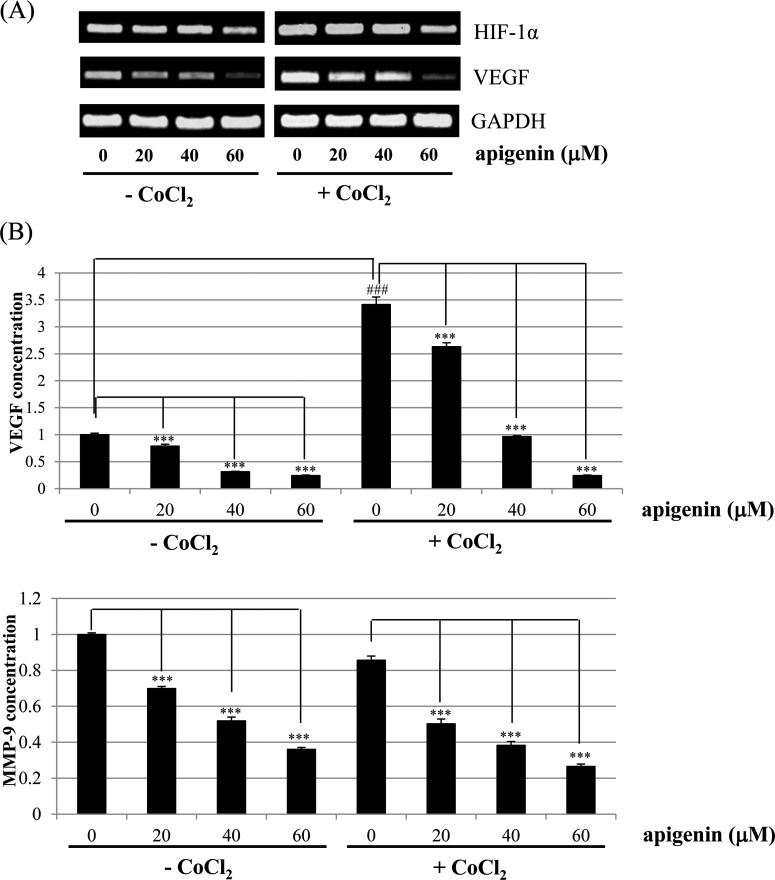
Effect of apigenin on the levels of HIF-1α and VEGF in BT-474 cells (**A**) BT-474 cells were treated with apigenin (0–60 μM) for 24 h in the presence or absence of CoCl_2_, and the mRNA levels of HIF-1α and VEGF were measured by RT-PCR. The data shown are representative of three independent experiments that gave similar results. (**B**) BT-474 cells were treated with apigenin (0–60 μM) for 24 h in the presence or absence of CoCl_2_, and the intracellular VEGF and MMP-9 concentration was measured by ELISA. Data are shown as the means of three independent experiments (error bars denote S.D.). *P<0.05, **P<0.01, ***P<0.001.

As shown in Figure 7(A), apigenin decreased mRNA levels of *HIF-1α* and *VEGF* that was slightly up-regulated by CoCl_2_. Moreover, ELISA assay indicated that apigenin strongly decreased the CoCl_2_-induced up-regulation of intracellular VEGF (Figure 7B). Apigenin also decreased intracellular MMP-9 level (Figure 7B). These results suggest that apigenin decreases the production of STAT3 target genes such as VEGF and MMP-9. These results also suggest that apigenin suppresses HER2-positive breast cancer cell growth rate by inhibiting the JAK-STAT3-VEGF signalling pathway.

### Effect of S3I-201 on STAT3 activation in BT-474 cells

Finally, we investigated whether the STAT3 inhibitor S3I-201 inhibits cell proliferation and STAT3 activation in BT-474 cells. As shown in Figures 8(A) and 8(B), S3I-201 decreased cell growth in a dose- and time-dependent manner. Furthermore, S3I-201 reduced the expression of p-STAT3, STAT-3 and VEGF (Figure 8C). These results demonstrate that STAT3 inhibition induces cell growth inhibition and represses the expression of oncogenic molecules. We also found that HIF-1α inhibitor (EF-24) and JAK inhibitor I induced cell growth inhibition (Figure 8D and 8E) in BT-474 cells. This suggests the involvement of HIF-1α and JAK pathways in the action of apigenin.

**Figure 8:**
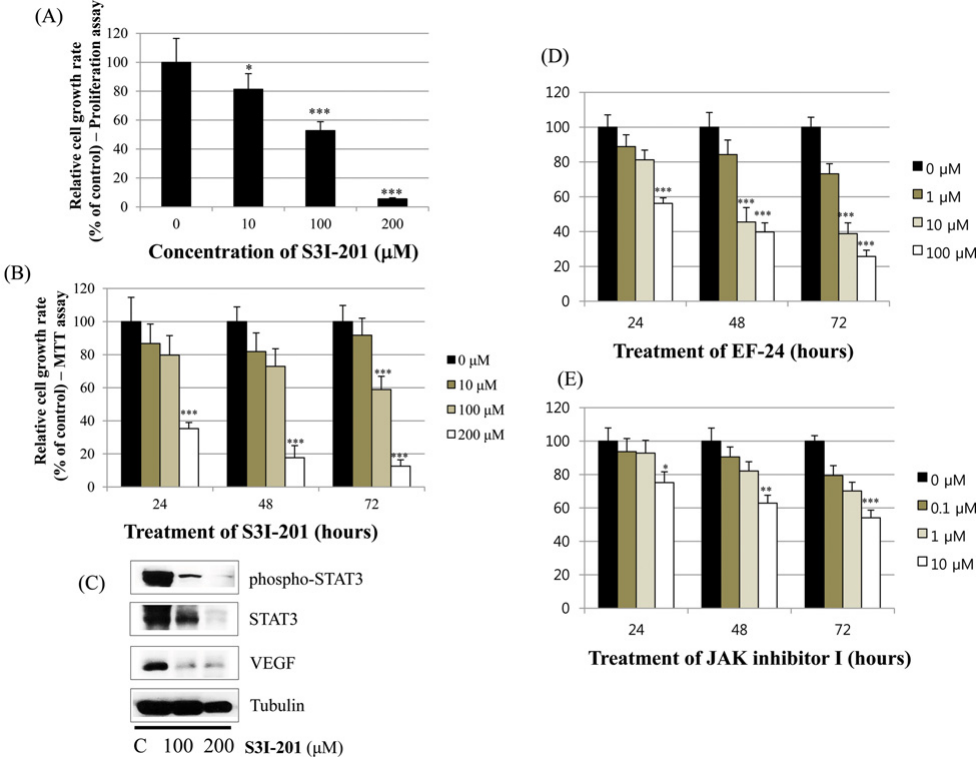
Effect of the STAT3 inhibitor S3I-201 on the growth of BT-474 cells (**A**) BT-474 cells were treated with different doses of the STAT3 inhibitor S3I-201 (0–200 μM). After 72 h, the cell viability was assessed using a cell proliferation assay. (**B**) BT-474 cells were treated with different doses of the STAT3 inhibitor S3I-201 (0–200 μM). The relative cell growth rate was measured by MTT assay after 24 h, 48 h and 72 h. The growth rate of the vehicle-treated cells was set to 100%, and the relative decrease in cell viability resulting from the S3I-201 treatment was expressed as a percentage of the control. Data are shown as the means of three independent experiments (error bars denote S.D.). *P<0.05, **P<0.01, ***P<0.001. (**C**) BT-474 cells were treated with the STAT3 inhibitor S3I-201 for 24 h. Whole-cell lysates were analysed by western blotting with anti-p-STAT3, anti-STAT3, anti-VEGF and anti-tubulin antibodies. The data shown are representative of three independent experiments that gave similar results. (**D**) (**E**) BT-474 cells were treated with different doses of the HIF-1α inhibitor EF-24 (0–100 μM) or JAK inhibitor I (0–10 μM). The relative cell growth rate was measured by MTT assay after 24 h, 48 h and 72 h. The growth rate of the vehicle-treated cells was set to 100%, and the relative decrease in cell viability resulting from the treatment was expressed as a percentage of the control. Data are shown as the means of three independent experiments (error bars denote S.D.). *P<0.05, **P<0.01, ***P<0.001.

## DISCUSSION

In the present study, we investigated the mechanism by which apigenin suppresses the growth of HER2-overexpressing breast cancer cells. The purpose of the present study is whether apigenin could serve as a useful compound to prevent or treat HER2-overexpressing breast cancer. Apigenin suppressed the growth of BT-474 cells in a dose- and time-dependent manner. Clonogenic survival assays revealed that apigenin decreased anchorage-dependent and anchorage-independent colony formation in a dose-dependent manner. These growth inhibitions were related with an increase in the sub-G_0_/G_1_ apoptotic population in BT-474 cells. Apigenin increased the number of apoptotic cells in a dose-dependent manner, as assessed by FACS analysis. Interestingly, apigenin did not induce apoptosis via intrinsic mitochondrial apoptosis pathway since the compound failed to regulate the levels of Bcl-2 and BAX, and did not reduce mitochondrial transmembrane potential (ΔΨm) maintaining red fluorescence. On the other hand, apigenin-induced apoptosis via caspase-dependent extrinsic apoptosis pathway showing the cleavage of caspases-8, -3, and PARP. Moreover, apigenin suppressed the cell growth even in the presence of caspase-8 inhibitor Z-IETD-*fmk* and the caspase-9 inhibitor Z-LEHD*-fmk*. These results indicate that apigenin contains a strong apoptotic activity. Caspases are a family of proteases that offer crucial links in cell regulatory networks related to inflammation and cell death (39). When activated, apoptotic caspases cause inactivation or activation of substrates, and the production of a cascade of signalling events permitting the controlled demolition of cellular components [39]. Dysregulation of caspases results in human diseases including cancer and inflammatory disorders [39]. Hence, the investigation of the caspase-dependent apoptotic pathway is important to prevent or treat breast cancer.

Apigenin increased the expression of active p53 (p-p53) suggesting that this compound suppresses HER2-overexpressing breast cancer cell growth via a p53-dependent manner. In agreement with our data, apigenin has been shown to induce G_1_ or G_2_/M arrest and apoptosis in human prostate carcinoma cells [40,41], human cervical carcinoma cells [42], and human hepatoma cells [43] through p53-dependent manner. The p53 tumour suppressor inhibits cellular proliferation by inducing cell-cycle arrest and apoptosis in response to cellular stresses including DNA damage, growth factor deprivation, hypoxia and oncogene activation [44,45]. p53-dependent apoptosis is produced by the caspase proteinases and related to pro-apoptotic proteins such as BAX, NOXA and PUMA [44].

Interestingly, apigenin reduced the expression of p-STAT3, p-JAK1 and p-JAK2 (upstream kinase of STAT3), and VEGF (STAT3 target gene) suggesting its negative regulation of STAT3 pathway in BT-474 cells. Elevated p-STAT3 expression by CoCl_2_ was also reduced by apigenin. Apigenin inhibited nuclear localization of STAT3 in the presence or absence of CoCl_2_ as revealed by immunocytochemistry. Apigenin inhibited the production of VEGF and MMP-9 as revealed by ELISA assay. The STAT3 inhibitor S3I-201 decreased the cell growth and expression of p-STAT3, STAT3 and VEGF in BT-474 cells. These results clearly indicate that apigenin induces growth-suppressive activity by inhibiting STAT3 signalling pathway. STAT3 is a transcription factor that regulates the gene expression in response to various cellular stimuli and plays an important role in cell growth and apoptosis. STAT3 usually acts as a tumour promoter, although its role as a tumour-suppressor has been previously reported [46,47]. STAT3 accelerates cell proliferation and angiogenesis, inhibits apoptosis, and drives invasion and metastasis [48–50]. STAT3 in melanoma tumours is associated with poor prognosis [48–50]. Constitutive STAT3 phosphorylation is mediated by several upstream kinases (Jak and Src) and is thought to be a key component of the oncogenic process [51,52]. Phytoestrogen (resveratrol) is known to inhibit STAT3 signalling and induces the apoptosis of malignant cells containing activated STAT3 [53]. The VEGF promoter contains various transcription factor binding sites, including sites for STAT3 [54] and HIF-1 [55]. The physical interaction of STAT3 with HIF-1 controls VEGF transcriptional activation by their binding to the VEGF promoter [56].

HER2-positive breast cancers show HER2 gene amplification or HER2 protein overexpression. HER2-positive breast cancers occupy 20–25% of invasive breast carcinomas [57]. HER2-positive breast cancers have a tendency to grow faster and are more likely to spread and relapse compared with HER2-negative breast cancers. HER2 is a member of the HER/ErbB2/Neu protein family, which also includes HER1/EGFR, HER3 and HER4. HER2 cross-talks with the ER signal transduction pathway [58], and its expression level can be regulated by ER. Our new finding of the present study is that apigenin induces caspase-dependent apoptosis through inhibition of STAT3 signalling pathway. In our study, we found that apigenin significantly inhibited the growth and induced apoptosis in HER2-overexpressing breast cancer cells. This indicates that apigenin could be a useful natural therapy that inhibits HER2-overexpressing breast cancer. Apigenin could be a promising target for the treatment and prevention of HER2-overexpressing breast cancer. The major questions remain about our study are whether apigenin overcomes drug resistance in the treatment of HER2-overexpressing breast cancer. This leads to further investigation.

## Abbreviations

AIF: apoptosis-inducing factor
Bcl-2: B-cell lymphoma 2
BAX: Bcl-2-associated X protein
ER: oestrogen receptor
HRP: horseradish peroxidase
PARP: poly(ADP-ribose) polymerase
PMSF: phenylmethylsulfonyl fluoride
SAv-HRP: streptavidin-conjugated horseradish peroxidase
STAT3: signal transducer and activator of transcription 3
TMB: tetramethylbenzidine
